# Stakeholder Perspectives on the Meaningful Integration of Clinical Informatics Interventions Using Patient-Reported Outcomes in Healthcare

**DOI:** 10.1055/a-2461-3027

**Published:** 2024-12-17

**Authors:** David Russell, Yashika Sharma, Andrew P. Ambrosy, Kelly Axsom, Janejira J. Chaiyasit, Margaret O. Cuomo, Christi Deaton, Anne J. Goldberg, Parag Goyal, Angel Guan, Fernanda C. G. Polubriaginof, Lucy McGurk, Alexander T. Sandhu, John A. Spertus, Meghan Reading Turchioe, David K. Vawdrey, Ruth Masterson Creber

**Affiliations:** 1 Department of Sociology, Appalachian State University, Boone, North Carolina, United States; 2 Columbia University School of Nursing, New York, New York, United States; 3 Department of Cardiology, Kaiser Permanente San Francisco Medical Center, San Francisco, California, United States; 4 Columbia University Vagelos College of Physicians and Surgeons, New York, New York, United States; 5 University of Cambridge School of Clinical Medicine, Cambridge, United Kingdom; 6 Weill Cornell Medicine, New York, New York, United States; 7 Memorial Sloan Kettering Cancer Center, New York, New York, United States; 8 Division of Cardiology and Stanford Prevention Research Center, Department of Medicine, Stanford University School of Medicine, Stanford, California, United States; 9 University of Missouri - Kansas City’s Healthcare Institute for Innovations in Quality and Saint Luke’s Mid America Health Institute, Kansas City, Missouri, United States; 10 Geisinger, Danville, Pennsylvania, United States

**Keywords:** patient-reported outcomes, qualitative, RE-AIM, equity, sustainability

## Abstract

**Background:**

Patient-reported outcomes (PROs) capture where patients are on their disease trajectory and can identify changes in health status from their perspective.

**Objectives:**

This study applied the equity and sustainability-informed RE-AIM framework (Reach, Effectiveness, Adoption, Implementation, and Maintenance) to gain insights into clinical informatics interventions for collection and use of PROs across health systems.

**Methods:**

A total of 14 health informatics and clinical professionals were interviewed about the development and use of PROs within their health systems and individual practices. Directed content analysis was performed to highlight patterns, similarities, and differences in stakeholder perspectives across RE-AIM domains.

**Results:**

The reach of clinical informatics interventions using PROs varied across clinical practices and settings based upon institutional commitment and support, integration of clinical information systems, and engagement with patients and families. Although interventions using PROs were viewed as effective for enabling focused conversations with patients and facilitating shared decision-making, barriers to adoption included licensing requirements associated with PRO instruments, lack of incentives for their use, limited integration of PRO results into electronic medical record systems, and poor support for patients with low technology and/or health literacy. Implementation of interventions using PROs was facilitated through training and support staff who aided clinicians with clinical workflow integration, availability of questionnaires in multiple languages, identifying thresholds and strategies for action, and presenting interpretable visualizations showing changes over time alongside significant clinical events. Maintenance of interventions using PROs was enabled through multimodal data collection approaches and data governance groups that evaluated organizational requests to track new measures.

**Conclusion:**

Initiatives to increase the reach of clinical informatics interventions using PROs will require health system investments into medical record system integration, education, and implementation support for clinicians and patients, and efforts to reach patient populations with language barriers or limited technology literacy.

## Background and Significance

Patient-reported outcomes (PRO) assess the status of a patient’s health directly from the patient. PROs capture domains of physical, social, and emotional health and can be used during both routine care and recovery from a major clinical event to provide a holistic picture of where patients are on their disease trajectory. PRO monitoring has shown benefits including increased survival and reduced financial difficulties in patients undergoing cancer treatment,^1–3^ improvements in symptom severity among patients with eczema,^4^ greater accuracy in health status assessment, and better ratings of clinicians’ understanding of symptoms in patients with heart failure.^5^ Studies also identify minimal clinically important differences in PRO for specific disease groups—i.e., thresholds of change that patients perceive as meaningful—which can help facilitate the interpretation of changes in scores over time. Such insights can facilitate more informed discussions with patients about changes in health.^6^ Research on PRO implementation supports their acceptability and usefulness by improving consistency in patient history-taking, facilitating focused and targeted patient–clinician conversations including triage, gaining a more accurate picture of the patient’s health status and trends over time, and gathering data for clinical decision-making.^7,8^

Despite these advances, researchers have identified barriers to integrating PRO into clinical practice. These include technical challenges with implementation of PRO collection and monitoring tools in medical record systems and perceptions among healthcare professionals that patients are resistant to completing PRO questionnaires, that PROs generate too much data to sift through or are not presented in readily interpretable ways (e.g., changes over time), and that reviewing PRO findings with patients will disrupt clinical workflows.^8–10^ Clinicians also harbor uncertainty about the benefits of using PROs and have shared fears that their use will divert attention from more acute problems.^8–10^ Clinicians also remain reluctant to use PROs due to limited guidance on when or how to incorporate findings into practice, hindering shared decision-making among patients and caregivers. Openness to PRO use is largely driven by institutional factors that either facilitate or restrict the integration of PROs into routine clinical care. Response rates to PRO questionnaires vary considerably and are impacted by health status, language proficiency, digital literacy, numeracy, and health literacy. These factors may hinder equitable reach across patient populations.^11,12^ Gaps in the literature include research that explores factors shaping the reach of clinical informatics interventions using PROs, approaches for integrating related technology applications into clinical workflows, and ways of implementing training and support to facilitate their use within clinical teams.^13^

To address these gaps, this qualitative study of health informatics and clinical professionals applied the RE-AIM (Reach, Effectiveness, Adoption, Implementation, and Maintenance) framework to gain insight into how health systems adopt, implement, and maintain clinical informatics interventions for collection and use of PROs. Specifically, we explored stakeholders’ perspectives on the reach of clinical informatics interventions using PROs across patient populations and representation across groups, their *effectiveness* in improving health outcomes and healthcare quality, *adoption* within health systems and clinical practices, barriers and enabling factors to *implementation*, and reinforcing factors that enable their *maintenance*.^14^ Insights were sought about contextual details surrounding implementation of clinical informatics interventions using PROs, as well as concerns of equity (i.e., when and where inequities might emerge or worsen during implementation) and sustainability (i.e., accessibility of interventions to underserved communities with fewer health-promoting resources).^14,15^

## Methods

### Study Design and Participant Recruitment

This study drew on qualitative interviews with health informatics and clinical professionals who were currently using, or sought to implement, clinical informatics interventions involvingPROs. Study participants were recruited using purposive and snowball sampling methods. An initial group of participants was identified by the research team based on their: (1) reputation as national leaders in clinical informatics interventions involving PROs; and/or (2) clinical practice expertise. Participants were approached via an e-mail message outlining the study objectives and research methods. This initial group of participants recommended additional contacts who fulfilled the study criteria and who were approached using the same method. Snowball sampling methods facilitated our identification of a wider network of relevant stakeholders and health systems than would have been reachable through direct connections alone. We did not set selection criteria for interviewees as to have had exclusively successful experiences with integrating clinical informatics interventions using PROs into their practices or health systems. This approach facilitated our gathering of participant perspectives across a range of implementation experiences. Interviewees included: (1) 10 clinicians (i.e., four cardiologists and six nurse practitioners) practicing at three health systems (i. e., Columbia University Irving Medical Center, Kaiser Permanente San Francisco Medical Center, Saint Luke’s Mid America Heart Institute), and (2) four health informatics professionals overseeing PRO development and integration at three health systems (i.e., Memorial Sloan Kettering Cancer Center, Geisenger, University of Utah Health). Soliciting stakeholder perspectives across six different health systems enhanced the external validity of our findings.

Three doctorally prepared researchers (R.M.C., Y.S., D.R.) conducted virtual interviews using a semi-structured interview guide aligned with the equity and sustainability-informed RE-AIM framework^15^ (Table 1). Interviewers had disciplinary expertise in nursing and sociology—and had conducted prior research on clinical informatics interventions involving PROs that informed our selection of frameworks and research questions. Interview questions were developed by five members of our research team (D.R., Y.S., R.M.C., M.R.T., P.G.) and were informed by previous qualitative research utilizing the RE-AIM framework to evaluate clinical informatics interventions involving PROs.^16^ Interview questions addressed the use and collection of PROs within practices and organizations (reach), perceived impacts of PROs (effectiveness), factors for collection of PROs (adoption), organizational supports and integration of PROs into clinical practice (implementation), and facilitators/barriers affecting use of PROs within clinical practices and the broader health system (maintenance). We allowed flexibility during our semi-structured interviews to ask probing and/or clarifying questions of participants that helped to elucidate their experiences with PROs in greater detail. We also ensured that all interviewees were asked similar questions under each domain of this framework. Verbal informed consent was obtained from participants to audio-record and transcribe their interviews, which lasted an average of 46.5 minutes (standard deviation = 8.1 minutes; range = 30 to 60 minutes). Informed consent processes involved sharing study objectives with participants, activities involved in participation, and ways in which the data from interviews would be used for the study. Audio recordings of interviews were created using built-in features of the conferencing software and were stored on a secure network drive accessible only to the research team. We emphasized the voluntary nature of these interviews and protections to maintain confidentiality. Participants were recruited until saturation was reached—defined as when further interviews yielded few additional insights into the properties and dimensions of RE-AIM domains related to PRO collection and monitoring.^17^ Study protocols were reviewed by the Institutional Review Board at Columbia University and determined to be exempt because they posed no more than minimal risk to study participants (Protocol#AAAU9136). We followed the Consolidated Criteria for Reporting Qualitative Research (COREQ; see Supplemental Table S1, available in the online version).^18^

### Data Analysis

Directed content analysis was performed iteratively over a series of eight meetings among three team members (D.R., Y.S., R.M.C.) to extend the RE-AIM framework to the study of clinical informatics interventions involving PROs.^15,16,19^ These members reviewed interview transcripts in their entirety to familiarize themselves with the data. During this stage of the analysis, text was highlighted in transcripts that illustrated components of the RE-AIM framework, including concerns about equity and sustainability. Excerpts pertaining to these initial coding categories were further analyzed to compare perspectives between health informatics professionals and clinicians. An analytic memo was created to further describe each of these coding categories. Gradually, this memo was refined into a report of study findings, complete with exemplar quotes labeled by interview number and stakeholder group (i.e., H—Health Informatics Professional; C—Clinician).

## Results

### Reach

The *reach* of clinical informatics interventions using PROs can be assessed by the quantity of patients impacted, the degree to which interventions extend to those with the greatest needs, and how representative patients receiving interventions are of the larger patient population.^14,15^ The reach of interventions using PROs varied across the health systems we studied. Health informatics professionals based at health systems with extensive reach of interventions using PROs attributed their wide use to institutional leadership, dedicated training and support staff, integration with clinical informatics systems, and engagement with clinicians, patients, and families (see Table 2). In contrast, clinicians practicing at health systems with lesser reach of interventions involving PROs described inconsistent approaches to their collection and monitoring across departments and divisions. Interviewees viewed multimodal approaches to PRO collection, including electronic questionnaires, follow-up phone calls, and in-person assessments, as beneficial to broadening the reach of these tools. However, variable response rates to PRO questionnaires were also attributed to gaps in reaching patients with lower literacy and those who do not speak English. Both clinicians and health informatics professionals highlighted the limited integration of translated questionnaires for non-English-speaking patients into patient portals of electronic health records. When translated questionnaires were not available, clinicians were reliant on more resource-intensive methods, including interpreters who could assist patients with translating and completing questionnaires. These methods were viewed as “time consuming” and often not conducted:

“*You would*… *I don’t do this. But we would have to have an interpreter sit down and fill out those questions [on Spanish language PRO questionnaires]. It’s very time consuming*.” [C2]

### Effectiveness

The *effectiveness* of clinical informatics interventions involving PROs is assessed by how well they achieve targeted outcomes, promote improvements in quality of life for patients, produce unintended consequences, and/or incur substantial costs for practices and patients.^14^ Health informatics professionals highlighted how PRO summaries can aid to identify at-risk populations, present clinicians with actionable tasks to manage risks with early intervention, and assist with recall of patient symptoms from previous clinical visits. Health informatics professionals distinguished PROs from other “*random [and unvalidated] patient-generated health data*” [H5]. Clinicians likewise viewed PROs as providing them with “*objective data*” [C1] that assisted plain-language communication with patients about changes in their health status between visits and facilitated referrals. For example, health informatics professionals at one health system described how the Patient Health Questionnaire (PHQ-9) was used to identify patients with depression and/or suicidal ideation and provide clinicians with decision support and best-practice recommendations for reaching them and connecting them to resources. Clinicians described how PROs illuminated changes in symptoms and functioning that might not otherwise be apparent in other unvalidated patient-generated health data, and facilitated more focused interactions with patients by encouraging lines of questioning about those changes and alternative treatment options. A nurse practitioner [C2] spoke about how worsening scores on the Generalized Anxiety Disorder 7-item (GAD-7) tool could accompany feedback from patients who share that they are “*feeling worse*” and be used to prompt a conversation about medications, while a cardiologist [C10] described how a significant change in the Kansas City Cardiomyopathy Questionnaire (KCCQ) summary score prompted additional questioning that uncovered a patient’s atrial fibrillation.

### Adoption

The *adoption* of clinical informatics interventions is captured by the absolute number, proportion, and representativeness of settings and persons who are willing to initiate a program. Factors for *adoption* of clinical informatics interventions involving PROs described by health informatics professionals and clinicians included (1) strategic priorities to address certain patient populations or disease groups, (2) licensing requirements for the use of PROs, (3) reimbursement mechanisms and other incentives to support collection of PROs, (4) alignment of PROs with quality metrics, (5) technology staff with willingness and ability to build questionnaires and troubleshoot challenges, and (6) an institutional environment where colleagues are willing to try new approaches, despite initial reservations. Illustrating how strategic priorities impacted adoption, one health informatics professional described how evidence that PRO monitoring led to improved survival outcomes among patients “*caught the attention*” [H7] of leaders at a large academic cancer center and motivated them to integrate the technology interventions from a PRO monitoring trial throughout their institution.^1,2^

“*There was a lot of investment that really started because of [trial research demonstrating improved cancer survival outcomes from PRO monitoring]. [This research sparked the] development of a simple technology solution that was kind of sitting on the side to support specific trials. The findings really caught the attention of the institution*.” [H7]

Clinicians discussed how insurance reimbursement for PRO collection could facilitate rapid adoption of PRO collection interventions. Additionally, both clinicians and health informatics professionals expressed that patients are willing to complete PRO questionnaires if they perceive their clinical team finds value in the information. Health informatics professionals discussed how the widespread adoption of pre-visit electronic COVID symptom questionnaires during the pandemic normalized PRO collection and increased patients’ comfort with portals. However, one cardiologist [C11] cautioned that without infrastructures in place and available staff to review PRO results, PRO collection alone, without appropriate responsiveness, will limit its meaningful use to improve clinical outcomes.

### Implementation

The *implementation* of clinical informatics interventions is shaped by barriers and enabling factors within clinical settings and larger healthcare organizations that support their use and delivery to patients and clinicians.^14^ Clinicians and health informatics professionals both highlighted how interventions involving PROs need to be tailored to specific disease conditions and thoughtfully integrated into clinical workflows and informatics systems in consultation with multiple stakeholders. Clinicians also highlighted the importance of integrating PROs directly into an organization’s medical record system, including questionnaire administration and result summaries. Stressing this point, a cardiologist [C10] highlighted how their organization chose to integrate PROs into its existing medical record over an alternative platform that may have provided more streamlined collection and attractive visualizations. Related to this point, clinicians argued in favor of implementation strategies that enable visualization of PRO findings over time and are presented alongside other aggregate patient data with annotations for clinical events. A cardiologist [C6], for instance, described how findings from the KCCQ could be presented for a patient over time, with points highlighted for procedure dates—e.g., valve replacement or cardiac resynchronization. The placement of PRO results within the medical record, including by directly embedding findings into clinical notes, was highlighted as a method for ensuring the results would be placed in front of clinicians while preparing for visits. However, stakeholders cautioned that presenting clinicians with a multitude of data can introduce challenges with seeing “*the forest through the trees*” [C8]. Clinicians expressed openness toward using PROs, but noted that given the burden required to collect and review them, data must be carefully selected and curated. To enhance the value of these data, clinicians suggested that PRO results should be translated into a clinically interpretable framework (e.g., defining meaningful score changes) that can be presented back to patients and clinicians to facilitate shared decision-making about treatment options.

Clinicians with less experience using PROs in their practice expressed uncertainty about the meaning of PRO scores, whether they reflected better or worse health status, thresholds for determining significant changes between assessments, and how to integrate findings into their clinical decision-making. Clinicians were also reluctant to take action based on PRO findings when they had reservations about available treatments. For instance, a nurse practitioner [C5] expressed hesitation to use the PHQ and GAD-7 due to perceptions that mental health resources were not easily accessible to patients and that available centers were impacted by staff turnover:

“*If we measure [PROs for mental health] and see change* … *worsening* … *we want to do something about it. But you know that is another, just a general boundary that we have right now ... access to mental health [services] ... finding covered providers that have good availability* … *there’s just so much turnover, in terms of mental health centers as well.”* [C5]

Implementation of clinical informatics interventions using PROs was facilitated by training and support staff who helped clinicians set up routines for using the tools in their practice, including note templates and alerts. One cardiologist [C10] emphasized how training extends to clinical support staff, including front-desk personnel, who should be instructed on rationales for PRO collection and why it is important that patients complete questionnaires prior to visits. PROs should be implemented in a manner that patients with less technology and health literacy are comfortable using them. Usability sessions with diverse patient populations should be conducted to understand item literacy and make changes when necessary to simplify language. Clinicians described how providing context to patients for interpreting PRO scores could aid in shared decision-making (e.g., that changes between visits reflect a loss of functioning impacting their social roles and relationships). Measures that would support implementation feasibility include: shortening PRO questionnaires, ensuring their translation in multiple languages, and providing guidance to clinicians about risk stratification and procedures candidacy.

### Maintenance

The *maintenance* of clinical informatics interventions is assessed by the extent to which organizations and practices sustain programs over time.^14^ In the absence of direct payer reimbursement (i.e., billing codes) for collection, scoring, interpretation, and use of PROs, a cardiologist [C6] highlighted how it can be difficult to quantify the return on investment in PROs. Rather than seeing these investments as revenue-generating ventures, like some specialty surgeries, clinicians and health informatics professionals both advocated for viewing interventions using PROs as playing important roles in the promotion of population health, for example, by monitoring post-acute patients for changes in health status and alerting clinicians to changes that need their attention. Clinicians noted that the adoption of a new electronic medical record system necessitated investments to ensure its user-friendliness and compatibility with PRO collection modalities (e.g., tablets and/or kiosks for PRO collection in the waiting room), accurate scoring of PROs, and presentation of clear and attractive visualizations to patients and clinicians. Health informatics professionals highlighted how governance structures, comprised of institutional leaders, clinicians, patients, families, and other professionals, supported the ongoing use and maintenance of clinical informatics interventions using PROs by discussing strategies for their collection, and fitting their interpretation and discussion with patients within clinical workflows, processes for reviewing results, and ways of translating findings into actions that support patients along their care journey.

“*We’ve developed governance structures to support [clinical informatics interventions using PROs], not just [to guide] development of the technology, but [to understand] how to bring that in a meaningful way to the clinic. There was a lot of partnership with clinicians*.” [H7]

Once established, these governance structures aided organizations with reviewing and deciding upon new PRO collection requests, balancing technology resources to build and implement questionnaires with the relative clinical value and/or burden that these measures bring to the clinical team. Governance structures also assisted with decisions on thresholds for changes in PROs that necessitate clinical alerts.

## Discussion

Advances in the development and validation of PROs have provided health systems with powerful tools to identify where patients are on their disease trajectory and evaluate patients’ responses to treatment. However, limited research has examined factors for the adoption, implementation, and maintenance of clinical informatics interventions using PROs across health systems—most previous work has focused on a single implementation site. Drawing on a sustainability and equity-informed RE-AIM framework, we interviewed health informatics and clinical professionals across six different health systems with variable implementation experiences to identify facilitators and barriers shaping their use of clinical informatics interventions involving PROs.^14,15^ Both groups of stakeholders saw these interventions as aiding with identifying significant health changes in patients and prompting investigation into their causes. However, their reach and adoption across health systems varied based on implementation (e.g., medical record system integration and presentation) and maintenance (e.g., input from governance boards). Together, these findings suggest opportunities for furthering the reach and meaningful use of interventions involving PROs.

Although the reach of clinical informatics interventions involving PROs was extensive in some health systems we studied, echoing previous findings that institutional leadership and technological support can facilitate PRO collection and monitoring,^20^ stakeholders also indicated gaps in reaching patients with lower literacy and/or whose primary language is not English. These gaps were attributed to a lack of translated PRO questionnaires and limited data collection strategies outside of pre-visit electronic questionnaires. The lack of multimodal PRO collection options available in some health systems may account for racial/ethnic disparities in completion rates.^21^ Improving representation and inclusivity in PRO collection necessitates greater involvement of patients who have been historically underserved by research into the development and implementation of PROs, as well as comprehensive and inclusive collection methods that are sensitive to the needs and circumstances of patients.^22^ Additional work is warranted to minimize respondent burden, to improve questionnaire administration formats, and to enhance the ease with which results are integrated into existing clinical workflows.^23^

Although the effectiveness and implementation of PRO initiatives within certain health systems was well-established, such as those focused on particular populations (e.g., patients with cancer), interviewees belonging to health systems serving broader populations of patients with a wider range of diseases described variable adoption of PROs, limited integration of PRO results into medical record systems and clinical workflows, and less clarity about how to use PRO findings in their day-to-day practice. These findings align with a recent national study, which noted variable adoption of PROs across physician practices, driven in part by chronic care management strategies (e.g., registries), screening for medical and social risks, and patient responsiveness.^24^ Future initiatives may follow the lead of one recent project, which detailed its approach to seamlessly integrating PROs into the EHR, to reduce complexity for patients and ensure that findings are displayed alongside other important patient information.^25^

Implementation barriers to clinical informatics interventions using PROs also included clinician ambiguity about PRO score meanings and thresholds of change that are considered significant. These barriers may be greater in cardiology, where research on minimal clinically important differences in PROs is ongoing. Clinicians within this area had less experience implementing clinical informatics interventions involving PROs and harbored uncertainty about how to integrate PRO summaries into their practice. Research in cancer care settings suggests that patients who reviewed PRO measure summaries with their clinicians found those discussions helpful and wanted to follow-up during future visits about changes in those measures; clinicians and staff meanwhile found PRO reports useful and easy to interpret and helpful for documenting symptoms.^26,27^ Interventions which use PROs to flag emerging patient concerns and engage with allied healthcare professions could enhance their impact on healthcare outcomes.^28^

### Limitations

The qualitative nature of this study did not allow us to precisely describe differences in reach and effectiveness of PROs across health systems. Although our sample reflects a range of perspectives from clinician and health informatics professionals across six U.S. health systems with variable degrees of PRO clinical informatics intervention integration, we were unable to interview patients as part of this research due to constraints on our sampling strategy. Research that foregrounds patients’ perspectives on clinical informatics interventions using PROs can help illuminate their perceived importance to patients, factors shaping their reach in patient groups with lower response rates, strategies for integration into patient portals, visualizations that enhance comprehension, and ways of strengthening conversations between clinicians and patients regarding concerning symptoms and health changes. Ethnographic research that focuses on patient and clinician discussions of PRO summaries may represent one method for deepening our understanding of these topics and identifying promising directions for incorporating PRO interventions into practice.

## Conclusion

Initiatives to increase collection and monitoring of PROs will require health system investments in medical record system integration, education and implementation support for clinicians and patients, and efforts to reach patient populations with language barriers and challenges with technology literacy. Although PROs can guide clinicians in engaging with patients in shared decision-making when used to facilitate conversations about health changes and treatment progress, this potential is conditional upon institutional commitments to expand their reach across patient populations through inclusive, multi-method collection modalities and seamless integration of PRO questionnaires and findings into medical record systems. Additional research is needed to better understand strategies for presenting PRO results to clinicians and patients, ways to support their interpretation, and to evaluate their cost-savings for health systems and impacts on patient-centered health outcomes. System-level incentives, such as reimbursement for PRO collection, may also help to accelerate their use and adoption.

## Supplementary Material

Supplementary file

## Figures and Tables

**Table 1:** Examples of semi-structured interview questions aligned with the RE-AIM framework

**REACH**

**Health Informatics Professionals:** *How are PRO measures being used across clinical care teams at [health system]?* *Do you have a sense of how widely these measures are used across [health system]?* *Are all populations equitably reached by PRO measures? Who is not reached and why?*

**Clinicians:** *Which PRO measures do you use in your clinical practice?* *Who do you administer PRO assessments to?* *Are all patients invited to complete PRO assessments, or just certain subgroups?* *Do you have a sense of how many actually complete their assessments and access their results?* *At which points in a patient’s care journey are you assessing PRO measures?* *How and when can you see these results?*

**EFFECTIVENESS**

**Health Informatics Professionals:** *Are PRO implementation and monitoring initiatives effective? For whom?* *Are the health impacts of PRO initiatives experienced equitably across all groups?*

**Clinicians:** *How has the integration of PRO measures impacted your clinical practice?* *Are those impacts experienced equitably across all groups of patients?* *Can you provide an example where you have used a PRO measure to support patient care?*

**ADOPTION**

**Health Informatics Professionals:** *From your perspective, why did your organization begin using PRO measures in patient care?* *What electronic tools have you used for the initial capture and ongoing monitoring of PROs?* *What have been some barriers and facilitators to different informatics tools?* *What are some ethical and equity considerations related to technology and PROs?*

**Clinicians:** *Why did you choose to begin using PRO measures in your clinical practice?* *What factors or people influenced your decision to use PRO measures in your clinical practice?* *What changes might be needed to facilitate adoption of PRO measures in your practice?*

**IMPLEMENTATION**

**Health Informatics Professionals:** *How does your organization support your use of PRO assessments in clinical practice?* *Can you share the institutional history of adoption of PRO measures?* *What strategies have been employed to overcome language and cultural barriers in the presentation of PRO data?* *What approaches have been effective in making digital PRO data more accessible and understandable to various stakeholders?*

**Clinicians:** *Can you walk us through how you make sense of, and interpret, PRO results for your patients?* *How do PRO results fit into your overall clinical picture for a patient?* *How do you evaluate PRO results in relation to a patient’s therapies and treatments?* *How does your organization support your use of PRO assessments in clinical practice?*

**MAINTENANCE**

**Health Informatics Professionals:** *In your view, has assessing PRO measures helped your organization provide better care?* *What are the facilitators and barriers that might affect the use of PRO measures?* *What factors do you think optimize the use of PRO assessments in practices within your organization?*

**Clinicians:** *In your view, has using PRO measures helped you provide better care for patients? Why or why not?* *What are the facilitators and barriers that might affect the use of PRO measures?* *What factors do you think optimize the use of PRO assessments in practices within your organization?* *What helps sustain a program of collecting PRO assessments of your patients?*

Abbreviations: PRO, patient-reported outcome; RE-AIM, Reach, Effectiveness, Adoption, Implementation, and Maintenance.

**Table 2:** Themes from directed content analysis of stakeholder perspectives on use and integration of patient-reported outcome measures

Domain	Health informatics perspective	Clinician perspective	Exemplar quotes
Reach	• PRO reach is expanded through institutional support, investments in support staff, integration with clinical informatics systems, and engagement with clinicians, patients, and families	• Limited reach of PROs within day-to-day clinical practice • Gaps in PRO reach arise due to delayed adoption of translated questionnaires for non-English speaking patients	*“Though these tools have been around for a long time ... I just don’t feel like they’re clinically, in the day-to-day, utilized as much.”* [C1] *“We’ve been pushing, you know, my patient population in my clinic is 50% Spanish speaking. So, I think there is huge equity consideration. So, I think that that is going to change in the near future, but unfortunately, it hasn’t changed quite yet.”* [C10] *“There is very little in other languages. Spanish is probably the second most needed language here . We just started translating some of the portal.”* [H5]
Effectiveness	• PRO aids with identifying at-risk populations and presenting actionable tasks to manage risks for early intervention • PRO can reduce discordances between patient and clinician health assessments, as well as challenges with recalling symptoms from prior visits	• PRO illuminates changes in patient’s status that might not otherwise be apparent in other health data • PRO facilitates focused interactions with patients by encouraging questioning about those changes and alternative treatment options	*“If the patient’s GAD-7 score has gone up ... usually the patient will also say, ‘Yeah. I’m feeling worse’; it goes hand-in-hand. It supports the data.”* [C2] *“[With] the PHQ ... Being able to catch patients who are answering to having suicidal tendencies ... Not only are we identifying it, but we are giving [clinicians] best practice recommendations of how they can help manage it. We’re not just collecting the data, we’re actually making [it] actionable.”* [H9]
Adoption	• PRO adoption is facilitated through licensing instruments, incentives for collection, and alignment with quality metrics	• Patients are willing to complete PRO questionnaires if they feel the clinical team will find value in the information	*“Cancer centers are where you will find the highest use of PROs, because those tools have been validated. They were first out of the gate. They have incentives around collecting PROs.”* [H5]
Implementation	• PRO should be integrated into the health system of record to facilitate use among clinicians • Support staff are helpful to aid clinicians with setting up PRO collection/monitoring workflow • Implementation feasibility is supported by shorter PRO questionnaires, translation in multiple languages, and guidance for clinicians • Patients and caregivers who sit on governance boards can speak to how PROs enable close symptom monitoring	• Clinicians expressed some uncertainty about how to integrate PRO findings into clinical workflows • Clinicians seek clear options for using PRO findings to advise treatment	*“If we measure it and if we see the change, the worsening of it, of course we want, as providers, we’re going to want to do something about it.”* [C2] *“Nursing is a huge partner for us to understand what does that mean to the clinical workflow? What does it mean in clinical documentation? We shouldn’t be asking questions that nobody is looking at. Who is reviewing it? Who is taking action?”* [H7] *“On the clinical side, we also have a robust training team... There is somebody, elbow-to- elbow, helping those clinicians get everything set up. They will see their note templates ... and they will know, for nurses, where do I go for alerts?”* [H7] *“We shortened measures to make them more feasible to implement in routine care . They can stratify patients into higher or lower risk, or define who would be a better candidate for therapy. They can be used in population health management to survey all patients with heart disease and identify those who have a lot of angina or a lot of heart failure symptoms.”* [C6] *“I found it quite unlikely that my heart failure colleagues would go to a separate system outside of [medical record system] to look up the results. That’s why we decided to focus on [medical record system] integration, rather than this slightly more flexible system that has better visualizations and is probably a little bit more streamlined for capturing data.”* [C10]
Maintenance	• It can be difficult to quantify the return on investment for PRO • Establishing clinician and technology governance groups can help review and balance new PRO collection requests	• Collecting PRO requires investments in multimodal and user-friendly ways of obtaining responses	*“We’ve had some patient-reported questionnaires in our system for the last several years, and the IT group has a system for deciding on, you know, incorporation of subsequent PROs.”*[C10]

Abbreviations: C, clinician stakeholder; GAD-7, General Anxiety Disorder-7; H, health informatics stakeholder; PHQ, Patient Health Questionnaire; PRO, patient-reported outcome measures.

## Clinical Relevance Statement

Incorporating PROs into clinical encounters has potential to increase their effectiveness by focusing on key lines of questioning around health changes and involving patients in shared decision-making about their disease progress and courses of treatment.

## Multiple Choice Questions

1. Based on the findings of the study reported in this article, which of the following statements best describes the *reach* of patient-reported outcome measures across health systems in the U.S.?
  - Stakeholders at all health systems described extensive reach of patient-reported outcome measures within their practices and organizations.
  - Stakeholders at all health systems described limited reach of patient-reported outcome measures within their practices and organizations.
  - The reach of patient-reported outcome measure collection and monitoring initiatives varied across stakeholders’ health systems.

**Answer:** The answer is c. The reach of patient-reported outcome measures varied across the health systems where stakeholder interviewees were based, with some health systems reporting extensive reach of these measures and others describing limited reach of measures across patients and practices.

1. Of the following, which was **not** described by interviewees as a driving factor for the adoption of patient-reported outcome measures at health systems?
  - Medical record systems with streamlined collection and attractive visualizations.
  - Fulfilling licensing requirements for the use of patient-reported outcome measures.
  - Technology staff to build patient-reported outcome measure questionnaires and troubleshoot challenges.
  - Reimbursement mechanisms and other incentives to support collection of patient-reported outcome measures.

**Answer:** The answer is a. Although streamlined collection and attractive visualizations can support implementation of patient-reported outcomes, factors for adoption of these measures described by stakeholders included strategic priorities to address certain patient populations or disease groups, licensing requirements for the use of PROs, reimbursement mechanisms and other incentives to support collection of PROs, alignment of PROs with quality metrics, technology staff with willingness and ability to build questionnaires and troubleshoot challenges, and fostering an institutional environment where colleagues try new approaches, despite initial reservations.
